# A rare schizophrenia risk variant of *CACNA1I* disrupts Ca_V_3.3 channel activity

**DOI:** 10.1038/srep34233

**Published:** 2016-10-19

**Authors:** A. Andrade, J. Hope, A. Allen, V. Yorgan, D. Lipscombe, J. Q. Pan

**Affiliations:** 1 Department of Biological Sciences, College of Life Sciences and Agriculture, University of New Hampshire, Durham, NH 03824, USA; 2 Brown Institute for Brain Science, Providence, RI 02912, USA; 3 Stanley Center of Psychiatric Research, Broad Institute of Harvard and MIT, 75 Ames Street, Cambridge, MA 02142, USA

## Abstract

*CACNA1I* is a candidate schizophrenia risk gene. It encodes the pore-forming human Ca_V_3.3 α1 subunit, a subtype of voltage-gated calcium channel that contributes to T-type currents. Recently, two *de novo* missense variations, T797M and R1346H, of hCa_V_3.3 were identified in individuals with schizophrenia. Here we show that R1346H, but not T797M, is associated with lower hCa_V_3.3 protein levels, reduced glycosylation, and lower membrane surface levels of hCa_V_3.3 when expressed in human cell lines compared to wild-type. Consistent with our biochemical analyses, whole-cell hCa_V_3.3 currents in cells expressing the R1346H variant were ~50% of those in cells expressing WT hCa_V_3.3, and neither R1346H nor T797M altered channel biophysical properties. Employing the NEURON simulation environment, we found that reducing hCa_V_3.3 current densities by 22% or more eliminates rebound bursting in model thalamic reticular nucleus (TRN) neurons. Our analyses suggest that a single copy of Chr22: 39665939G > A *CACNA1I* has the capacity to disrupt Ca_V_3.3 channel-dependent functions, including rebound bursting in TRN neurons, with potential implications for schizophrenia pathophysiology.

*CACNA1I* encodes the pore-forming hCa_V_3.3 α1 subunit, one of three major Ca_V_3 voltage-gated calcium (Ca_V_) channels that contribute to low threshold T-type currents. Compared to Ca_V_ channels, Ca_V_3 have low voltage-activation thresholds, rapid inactivation, and slow closing rates^1^. Of the three-member Ca_V_3 family, Ca_V_3.3 channels have the most depolarized activation thresholds; and they open as well as inactivate and close slowly^2^.

Ca_V_3.3 channels are expressed in a limited subset of neurons including GABAergic neurons of the thalamic reticular nucleus (TRN) where they support oscillatory activity essential for sleep spindle generation^3^,^4^,^5^,^6^. In TRN neurons, Ca_V_3.3 channels are mostly inactivated at resting membrane potentials and mediate the rebound bursting upon transient membrane hyperpolarizations^7^,^8^. Rebound bursting in TRN neurons is largely absent in mice lacking Ca_V_3.3 channels, and sleep spindle generation is disrupted^6^,^9^. Sleep spindle abnormality, as well as altered patterns of neuronal activity in the thalamus, are found in people with schizophrenia, suggesting thalamocortical network dysfunction^10^,^11^,^12^,^13^,^14^.

Genetic analyses of large patient cohorts have identified loci associated with the risk of mental illnesses including schizophrenia, autism spectrum disorder and bipolar disorder^15^,^16^,^17^,^18^,^19^. Several of these candidate risk genes encode proteins involved in calcium signaling, including voltage-gated calcium channel subunits (*CACNA1C*, *CACNB2* and *CACNA1I)*, that may ultimately converge on a common disease mechanism^17^,^19^,^20^,^21^,^22^,^23^,^24^. Biological insight derived from psychiatric-associated common genetic variations is currently limited to a few studies^25^,^26^,^27^,^28^,^29^; causative risk alleles can be difficult to localize precisely within GWAS loci, and many map to non-coding regions of as yet undefined functions^25^,^30^. Recently, two rare, *de novo* missense variations, Chr22: 39659492C > T and Chr22: 39665939G > A, were identified in *CACNA1I* by exome sequencing of trio samples that include 105 schizophrenia probands. *CACNA1I* was the only gene in this cohort to harbor missense variations in more than one proband^31^. The resulting amino acid alterations, T797M and R1346H (numbered according to NM_021096 but equivalent to R1311H NM_001003406^31^), are predicted to reside in extracellular regions of domains II and III of hCa_V_3.3. *CACNA1I* was validated as a candidate schizophrenia risk gene, in a genome wide association study by the Schizophrenia Working Group of the Psychiatric Genomics Consortium as one of 108 independent genomic loci that exceed genome-wide significance^19^. These studies emphasize the need for functional studies of rare missense, exome variations in *CACNA1I* that associate with schizophrenia risk^25^,^31^,^32^.

Here we use biochemical and electrophysiological assays in the HEK293 cell expression system, to show that R1346H hCa_V_3.3 variant has reduced glycosylation and cell surface expression compared to hCa_V_3.3 wild-type, without altered biophysical properties. In contrast, T797M did not affect hCa_V_3.3 channel properties in our assays. Employing NEURON to simulate TRN neuron excitability, we show that R1346H disrupts rebound bursting mediated by Ca_V_3.3 channels.

## Materials and Methods

### Mutagenesis

T797M and R1346H were introduced on an SbfI-HindIII fragment of FLAG-tagged WT hCa_V_3.3 cDNA (Origene, RC219179) and sub-cloned into pcDNA5/FRT/TO vector (Thermo Fisher Scientific) to generate full-length FLAG-tagged hCa_V_3.3 cDNAs. Mutagenesis primers were for T797M: 5′-CACGGACACTGGAGACATGGTGCCCG/5′-TCGGGCACCATGTCTCCAGTGTCGGTG, and for R1346H: 5′-GCAACATCACCAACCACTCGGACTGC/5′-GCCATGCAGTCCGAGTGGTTGGTGAT. All constructs used in this study were sequence verified.

### Single copy cDNA, stable integration in HEK293 cells

The Flp-In T-REx system selects for clones containing single copy integration of cDNAs thereby reducing variability in Ca_V_3.3 protein expression levels among cells and across cell lines. Parental Flp-In T-REx HEK293 cells (Thermo Fisher, R78007) were transfected with pcDNA5/pFRT/TO vectors containing WT, T797M or R1346H hCa_V_3.3 cDNAs, and pOG44 vector encoding FLP recombinase (Thermo Fisher, K650001) in 1:9 ratio using Lipofectamine 2000 (Invitrogen). Cells were cultured in DMEM/F12 supplemented with 10% FBS. Single cDNA insertion events were selected after two weeks in 200 μg/mL hygromycin B (Invitrogen) and resistant polyclonal cell lines (10–20 colonies) expanded and maintained with 200 μg/mL hygromycin B and 15 μg/mL blasticidin S (Invitrogen). hCa_V_3.3 channel expression was induced by 1 μg/mL doxycycline and all analyses (including electrophysiology) were performed 72 hours after induction (unless otherwise indicated). At 72 hours Ca_V_3.3 channel protein levels plateaued. cDNA integration in each cell line was validated with Sanger sequencing of the insertion locus.

### Reverse transcription and quantitative PCR

Total RNA from each cell line was harvested using RNAeasy Plus (Qiagen). 10 μg of total RNA was reverse-transcribed and cDNA synthesized using random hexamer priming (Transcriptor cDNA synthesis, Roche). FastStart Universal SYBR Green 2X Master Mix (Roche) was used to perform quantitative PCR for hCa_V_3.3 (*CACNA1I*: *5*′*CAATGGACTGGATGCTGTTG/5*′*ATCCAGGGGTTGTGGTTG*) and β-actin (*ACTB: 5*′*CCAACCGCGAGAAGATGA/5*′*CCAGAGGCGTACAGGGATAG*). *CACNA1I* mRNA in each cell line was analyzed by the relative quantitation of gene expression method and using ACTB that encodes β-actin as the reference control gene (ΔΔCt method)^33^. Threshold amplification cycle (CT) values were obtained for target (*CACNA1I*) and internal control (*ACTB*) to calculate ∆CT (CT target–CT reference), and ΔΔCt calculated before and after induction of *CACNA1I* by doxycycline treatment. We carried out 3–4 technical replicates, from three independent cell culture and dox-induction step (biological triplicates). The biological variability in our RT-qPCR experiments stems primarily from different overall levels of mRNA induction across biological replicates.

### Immunoblotting

Whole cell lysates of Flp-In T-REx HEK293 cells expressing WT, T797M or R1346H hCa_v_3.3 channels were prepared as previously described^34^. Electrophoresis samples were prepared in 4x Laemmli buffer (Bio-Rad) and incubated at room temperature for 20 minutes before SDS-PAGE (3–8% tris-acetate gel, Life Technologies). Antibodies: mouse anti-FLAG (1:1000, Sigma F1804); rabbit anti-GAPDH (1:1000, Cell Signaling Technology 14C10); mouse anti-β-actin (1:50000, Sigma A5441); rabbit anti-pan-cadherin (1:1000, Life PA5-17526). Anti-GAPDH or anti-β-actin signals were used as reference signals to normalize across preparations and also as gel loading controls.

### Cell-Surface Biotinylation

hCav3.3 protein expression was induced in transformed Flp-In T-REx HEK293 cells with 4 day treatment of 1 μg/mL doxycycline. Cells were then washed three times with *solution A* (see below) and incubated with 1.5 mg/mL EZ Link Sulfo-NHS-SS Biotin (Life Technologies) for 20 min at 4 °C. The labeling reaction was quenched with 3 × 5 min BSA/L-lysine solution, and the cell preparation washed twice at 4 °C for 5 min with *solution A*. Cells were lysed on ice with 200 μL of PB/SDS and 1 mL of PB/Triton, sonicated for 5 min at 30 on/30 off intervals in a Bioruptor bath sonicator (Diagenode), and centrifuged at 13000 rpm for 15 minutes. Biotinylated lysates were incubated with NeutrAvidin beads (Thermo Fisher Scientific) under constant rotation for 2 hours, centrifuged at 2000 rpm for 30 s, and then beads were washed sequentially with 2 × 1 mL PB/Triton, 2 × 1 mL PB/Triton/600, and 2 × 1 mL of PB. Protein bound to NeutraAvidin beads was eluted for 20 minutes at RT°C with 2x Laemmli buffer (Bio-Rad) and β-mercaptoethanol. Input and eluate samples were analyzed by immunoblotting. *Solution A*: 7.74 mM Na_2_HPO_4_, 2.26 mM NaH_2_PO_4_, 2.7 mM KCl, 137 mM NaCl, 1 mM CaCl_2_, 0.5 mM MgCl_2_. *BSA/lysine quenching solution*: 0.5% BSA, 50 mM L-lysine-H_2_O in PBS/Ca/Mg, pH 7.4; PB: 7.74 mM Na_2_HPO_4_, 2.26 mM NaH_2_PO_4_, 5 mM EDTA, 5 mM EGTA, 100 mM NaCl, 10 mM Na_4_P_2_O_7_, 50 mM NaF, 1 mM Na_3_VO_4_; PB/SDS: PB, 1% SDS; PB/Triton: PB, 1% Triton; PB/Triton/600: PB,1% Triton, 600 mM NaCl.

The protein measurements for T797M and R1346H channels have similar dispersion, and scales proportionally to the absolute values. The coefficient of variation (CV) for whole cell protein level for R1346H is 40% compared to 31% for T797M. Using 1000 bootstrapping samples we estimated that the 95% confidence interval for T797M CV is (0.21,0.42), while the 95% CI for R1346H CV is (0.26, 0.54). The 95% confidence interval for the difference in CV between T797M and R1346H is (−0.26, 0.09)–not different from 0–indicating that the data dispersion between T797M and R1346H is statistically equivalent.

### Deglycosylation

Deglycosylation was performed on input or eluate samples with Protein Deglycosylation Mix (New England Biolabs). Briefly, 60 μg of total protein (input) or biotin labeled membrane protein (eluate) was denatured in Glycoprotein Denaturing Buffer for 20 minutes at RT. The denatured protein was then treated with either buffer alone (control) or with the Deglycosylation Enzyme Cocktail (PNGase F, *O*-Glycosidase, Neuraminidase, Galactosidase, and β-N-acetylglucosaminidase) for 1 hour at 37 °C in a solution of 50 mM sodium phosphate and 1% NP-40. Samples were analyzed by immunoblotting.

### Conventional electrophysiology

Voltage-gated calcium currents (Ca_V_) were only resolved in Flp-In T-REx HEK293 cells after, and not before, doxycycline induction of wild-type or mutant hCa_V_3.3 cDNA. Whole-cell patch clamp recordings were used to compare hCa_V_3.3 channel currents in doxycycline treated Flp-In T-REx HEK293 cells carrying a single copy of exogenous wild-type or mutant hCa_V_3.3 cDNA. Recordings were performed as previously reported^35^,^36^. The macroscopic Ca_V_3.3 currents recorded in this expression system originate from the gating of Ca_V_3.3 channels: we have never recorded Ca_V_ currents in untransfected tsA201, HEK293 or Flp-In T-REx HEK293 cells under the recording conditions used in this study (see also refs 35,37, 38, 39). Wild-type macroscopic Ca_V_3.3 currents shown here have all the properties of Ca_V_3.3 currents and the peak current is about 60 pA/pF, equivalent to ~ 700–750 pA, with only 2 mM calcium as the charge carrier. The properties of the macroscopic as well as single Ca_V_3.3 channel currents are completely consistent with previously published data e.g.^2^,^40^.

Whole-cell external recording solution contained: 2 mM CaCl_2_, 10 mM HEPES, 140 mM NaCl, pH adjusted to 7.2 with NaOH and the intracellular pipette solution contained: 126 mM CsCl, 10 mM EGTA, 1 mM EDTA, 10 mM HEPES, 4 mM MgATP, pH 7.2 with CsOH. Whole-cell hCa_V_3.3 currents were evoked by square step depolarizations from a holding potential of −100 mV. Currents were leak subtracted online using a P/−4 protocol. Currents recorded with Axopatch 200B amplifier (Molecular Devices, LLC) were sampled at 20 kHz and filtered at 2 kHz. For single-channel recordings, we used the HEK293-derived cell line tsA201 transiently expressing wild-type or mutant hCa_V_3.3 cDNA for 48 hrs. We used a pipette solution for single channel recording which is optimized to isolate Ca_V_ currents: 110 mM BaCl_2_, 2 mM CsCl, 10 mM HEPES, pH adjusted to 7.2 with Ba(OH)_2_ and the extracellular bath solution contained: 135 mM potassium aspartate, 10 mM EGTA, 5 mM HEPES, 5 mM KCl, pH adjusted to 7.2 with KOH (see refs 35,37, 38, 39). The high potassium extracellular solution effectively clamps the membrane potential to 0 mV eliminating contributions of the membrane potential to the transmembrane patch voltage. To reduce electrode capacitance in single channel recording, pipettes were coated with Sylgard 184 (Dow Corning, Co) and polished to resistances of 5–8 MΩ. Single channel currents recorded using Axopatch 200B amplifier (Molecular Devices, LLC) were sampled at 10–20 kHz and filtered at 1 kHz. Clampfit10 software (Molecular Devices, LLC) was used for single channel analyses. Leak subtraction was performed offline using a noiseless stimulated null trace. All recordings were obtained at room temperature.

### Properties of single Ca_V_3.3 channel currents

The estimated reversal potential for current flow through single Ca_V_3.3 channels was positive to 0 mV, the single channel conductance estimated from the single channel current voltage relationship was consistent with single channel conductance measurements for Ca_V_3, and Ca_V_3.3 specifically e.g^2^,^40^; single Ca_V_ channel currents exhibited classic slow closing kinetics during the repolarization step^2^; and we have never resolved single Ca_V_ channel currents in tsA201 cells in un-transfected cells^35^,^37^.

### Experimental Design

All data were acquired without knowledge of clone identity, all analyses were done before revealing clone identity, and the experimenter interleaved recordings from cells expressing different clones on each experimental day. To compare current amplitudes across cells, we converted to current density (pA/pF) to normalize for cell size. In high throughput electrophysiology, cell capacitance is not measured but the much larger sample sizes (>100) add statistical power and compensate for the relatively small variation in size among HEK293 cells. Peak currents evoked by depolarizing pulses were used to generate current voltage relationships. These were fit with the sum of a Goldmann-Hodgkin-Katz flux equation (ϕ) and an exponential function to model the shape of inward and outward unitary conductance, multiplied by a single Boltzmann function to summarize gating.


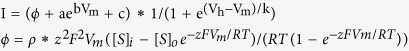


The following were held constant: R, the gas constant; F, Faraday’s constant; [S]_i_, internal Ca^2+^ (100 nM); [S]_o_, external Ca^2+^ (2 mM); z, valency of permeant ion (2); T, temperature (18 °C). We measure total whole-cell capacitance and estimate cell size using 1 μF/cm^2^.

### High throughput electrophysiology

Recordings from doxycycline-induced Flp-In T-REx HEK293 cells expressing hCav3.3 channels were performed using Ion-works Barracuda by ChanTest, Charles River^41^. Briefly, cells were harvested, washed and re-suspended in HEPES-buffered physiological saline before adding to the single-hole 384-well patch plate. External recording solution contained: 137 mM NaCl, 4 mM KCl, 7 mM CaCl_2_, 1 mM MgCl_2_, 10 mM HEPES, and 10 mM glucose, pH adjusted to 7.4 with NaOH. The internal solution contained 90 mM CsF, 50 mM CsCl, 2 mM MgCl_2_, 0.5 mM EGTA, and 10 mM HEPES, pH 7.2 adjusted with CsOH. Membrane currents were recorded with on-board patch clamp amplifiers, after establishing whole-cell configuration using amphotericin B (100 μg/ml). Peak current amplitudes measured from cells expressing hCa_V_3.3 WT, T797M and R1346H were fit to a bimodal distribution comprising two log-normal functions (sub-distributions) of different scale and shape factors. Descriptors of interest, the Bernoulli parameter and the median of the larger sub-distribution were determined by finding the parameters that maximize the likelihood of the data (maximum-likelihood estimates). To determine confidence intervals, a bootstrap procedure was used: the fitting algorithm was repeated for each resampling of the data.

### Simulations of TRN neuron activity in NEURON environment

We used the Huguenard lab model of TRN neuron bursting^7^ in the NEURON simulation environment (Accession number 17663, ModelDB^42^) and adapted the code into Python for analysis. We used a detailed, 80-compartment model of the dendritic arbor of a TRN neuron, with Ca_V_3.3 currents primarily concentrated in distal dendrites. Dendritic location of Ca_V_3.3 currents best captures the native firing pattern of TRN neurons^7^. All parameters were set at the default values except we varied the maximum conductance of Ca_V_3.3 channels between 40–120%^7^. Simulations of membrane voltage responses at soma were recorded during a series of 200 ms hyperpolarizing (−0.1 to −0.4 nA) or depolarizing (0–0.4 nA) current steps. The temperature was set at 36 °C.

## Results

### hCa_V_3.3 expression is disrupted by R1346H

**Figure 1:**
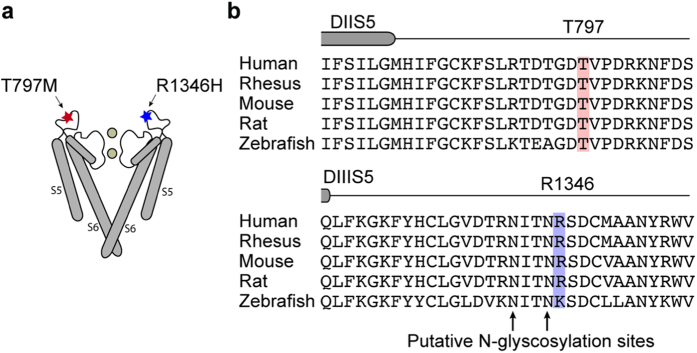
hCa_V_3.3 variants, T797M and R1346H, are predicted to be extracellular. (**a**) Approximate locations of T797M and R1346H in extracellular loops between transmembrane helices S5 and S6 in domains II and III of hCa_V_3.3. Figure highlights only S5 and S6 helices (S1–S4 missing), and pore regions of only two of four domains of a generic voltage-gated ion channel. (**b**) Amino acid sequences of Ca_V_3.3 aligned for five vertebrates for regions I774 and S806 (upper), and Q1326 and V1357 (lower). Numbering according to NM_021096. The end of transmembrane helices S5 in domains II and III are marked (DIIS5, DIIIS5). Sequence alignment illustrates the high degree of conservation of amino acids in Ca_V_3.3 surrounding T797 and R1346. Two putative N-glycosylation sites at asparagines 1345 and 1342 are marked (arrows; lower).

**Figure 2:**
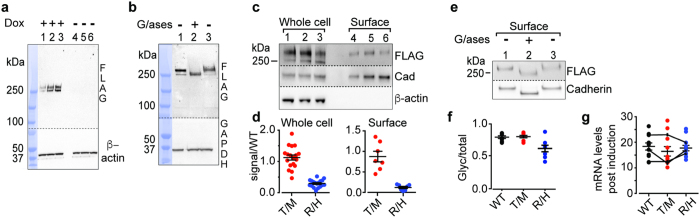
R1346H affects surface expression of hCa_V_3.3. All membranes were cut in two; upper membranes were probed with anti-FLAG to measure hCa_V_3.3 levels and lower membranes with control antibodies. (**a**) Anti-FLAG signals from Flp-In T-REx HEK293 cell lysates after induction for 24, 48, and 72 hrs with (lanes 1–3), and without (lanes 4–6), 1 μg/ml doxycycline. (**b**) Anti-FLAG signals in whole cell lysates from cells expressing WT-hCa_V_3.3, untreated (1), with glycosidase (2), and same as (2) but lacking glycosidase (3). (**a**, **b**) Compiled figures from 4 digital images of the same gel. Protein ladder images are juxtaposed to the immunoblots; ladder lane is colored in blue. Dotted lines indicate the spliced borders of two immunoblots. (**c**) Anti-FLAG hCav3.3 levels in whole cell lysate (1–3) and biotin-surface fraction (4–6) from cells expressing WT (1, 4), T797M (T/M) (2, 5), and R1346H (R/H) (3, 6). (**d**) Anti-FLAG signal in whole cell and biotinylated (surface) preparations from cells expressing T/M and R/H shown relative to WT and normalized to controls (cadherin and β-actin). Mean ± SE values for T/M were 1.12 ± 0.08 (n = 18, whole cell) and 0.87 ± 0.13 (n = 7, surface); for R/H were 0.29 ± 0.03 (n = 18, whole cell) and 0.12 ± 0.03 (n = 7, surface). Coefficient of variation: T/M, 31% and R/H, 40% (1000 samples bootstrapping). (**e**) Anti-FLAG signals in biotinylated surface protein from cells expressing WT-hCa_V_3.3, untreated (1), glycosidase exposure (2) and, conditions same as (2) but lacking glycosidase (3). (**f**) Average fraction of upper MW band relative to total hCa_V_3.3 for WT: 0.80 ± 0.02 (n = 8); T/M: 0.80 ± 0.02 (n = 8); and R/H: 0.62 ± 0.05 (n = 8) from data in (**c**,**d**). (**g**) RT-qPCR analysis of hCa_V_3.3 mRNA 72 hr after doxycycline induction expressed as fold-change relative to non-induced. Each individual point is a separate qPCR measure for 3 biological replicates and 3–4 technical replicates. Lines connect the relative levels of mRNA for three genotypes within each biological experiment. Data do not violate D’Agostino-Pearson test for normality.

We set out to test if two rare schizophrenia risk variations, identified in *CACNA1I* from exome sequencing of trio samples^19^, disrupt Ca_V_3.3 channel function as assessed in HEK cell line expression systems. Figure 1a,b illustrate the approximate location of amino acids T797 and R1346 in putative extracellular loops that link transmembrane helices 5 and 6 in domains II and III, respectively. We used Flp-In T-REx HEK293 cells that harbor inducible, single-copy stable integration of cDNAs encoding C-terminus FLAG-tagged WT, T797M, or R1346H, to investigate hCa_V_3.3 function. These cells do not express endogenous Ca_V_3.3 channels. The anti-FLAG Ca_V_3.3 signal was only induced following exposure to doxycycline (Fig. 2a, lanes 1–3) and absent in non-induced cells (Fig. 2a, lanes 4–6). Anti-FLAG Ca_V_3.3 signal increased in whole cell lysates prepared from stably transformed cells during 3 days of exposure to doxycycline, and plateaued 72 hours post induction (Fig. 2a, lanes 1–3).

WT hCa_V_3.3-FLAG protein isolated from whole cell lysates migrates as a doublet at ~250 kDa and >250 kDa (Fig. 2a). The >250 kDa band is glycosylated based on the shift to ~250 kDa after exposing cell lysates to glycosidases (Fig. 2b). We compared total hCa_V_3.3-FLAG protein levels in lysates from cells expressing WT, T797M and R1346H and found that the hCa_V_3.3-FLAG signal in cells expressing R1346H was reduced significantly compared to WT (Fig. 2c, compare lanes 1 and 3; Fig. 2d *Whole cell*, F(2,53) = 89.43, *p* < 0.0001, one-way ANOVA followed with Dunnett’s test, *p*_WT,R1346H_ < 0.0001). The relative level of the >250 kDa glycosylated hCa_V_3.3 signal, as a fraction of the total hCa_V_3.3 signal, was significantly reduced in cells expressing R1346H compared to WT controls (Fig. 2f, F_(2,23)_ = 10.04, *p* = 0.014, one-way ANOVA with Dunnett’s post test, *p*_WT, R1346H_ = 0.0439). By contrast, the total hCa_V_3.3 signal, and the relative abundance of the glycosylated >250 KDa signal from cells expressing T797M were not significantly different from WT (Fig. 2c, lanes 1 and 2; Fig. 2d, *whole cell*, one-way ANOVA followed with Dunnett’s test, *p*_WT,T797M_ = 0.2491; Fig. 2f, one-way ANOVA and Dunnett’s test, *p*_WT, T797M_ = 0.8318).

Biotinylated, surface hCa_V_3.3 isolated from cells expressing WT, T797M and R1346H migrates as a single, glycosylated band at >250 kDa (Fig. 2c, lane 4–6). The biotinylated R1346H Ca_V_3.3 signal was significantly lower compared to WT (Fig. 2d, *surface*, lane 4 and 6, F(2, 20) = 40.78, *p* = 0.0005, one-way ANOVA and Dunnett’s test, *p*_WT,R1346H_ < 0.0001), but biotinylated T797M hCa_V_3.3 was not distinguishable from WT (Fig. 2d, *surface*, lane 4 and 5, one-way ANOVA and Dunnett’s test, *p*_WT, T797M_ = 0.5469). Following glycosidase treatment, the surface, biotinylated WT hCa_V_3.3 signal shifted to a lower molecular weight (Fig. 2e), suggesting that the major fraction of surface hCa_V_3.3 is glycosylated. We analyzed Ca_V_3.3 mRNA levels in Flp-In T-REx HEK293 cells from three different induced cell cultures, and within each experiment quantified mRNA levels 3–4 times. We found no differences among cells expressing WT, R1346H, and T797M hCa_V_3.3 (Fig. 2g).

Our data demonstrated that hCa_V_3.3 protein levels are substantially lower in cells expressing R1346H compared to WT and T797M. The glycosylated R1346H hCa_V_3.3 protein fraction (>250 kDa) is preferentially reduced relative to WT (Fig. 2f), suggesting that R1346H might interfere with glycosylation and surface trafficking of hCa_V_3.3.

**Figure 3:**
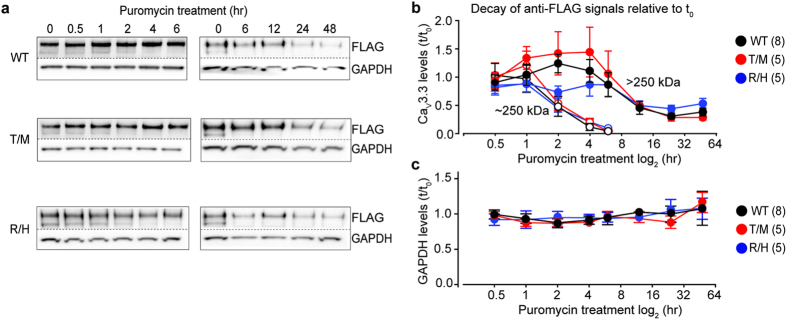
Glycosylated and non-glycosylated hCa_V_3.3 signals have different decay time courses after inhibition of protein translation. (**a**) Anti-FLAG signals from total cell lysates at different time points following exposure to 0.8 μg/mL puromycin from cells expressing WT, T797M (T/M) and R1346H (R/H). All membranes were cut in two; upper membranes were probed with anti-FLAG to measure hCa_V_3.3 levels and lower membranes with anti-GAPDH for normalization. (**b**) Time course of anti-FLAG hCa_V_3.3 > 250kDa (closed symbols) and ~ 250kDa (open symbols) signals normalized to GAPDH and represented relative to pre-puromycin levels for WT (black), T/M (red) and R/H (blue). The >250 kDa glycosylated signals were similar among WT, T/M and R/H except at the 2 hr time point. Mean ± SE at 2hr for WT: 1.42 ± 0.10 (n = 8); T/M: 1.32 ± 0.11 (n = 5); and R/H: 0.85 ± 0.17 (n = 5), at 48 hr for WT: 0.36 ± 0.08 (n = 8); T/M: 0.27 ± 0.06 (n = 5); and R/H: 0.50 ± 0.12 (n = 5). Mean ± SE are shown for each time point. For all analysis, results shown represent at least three experimental replicates and at least two technical replicates. Data do not violate D’Agostino-Pearson test for normality, and comparisons were analyzed by one-way ANOVA with Dunnett’s post hoc test. (**c**) GAPDH levels during puromycin treatment. The level of GAPDH at each time point is normalized to GAPDH level at time 0 for each condition. Each data point represents mean ± SE for three separate cultures.

To compare rates of hCa_V_3.3 protein decay in cells expressing WT, T797M and R1346H, we treated cells with 0.8 μg/mL puromycin to inhibit protein translation and measured levels of hCa_V_3.3 protein at different time points. At this concentration and duration of exposure, puromycin had no obvious cellular toxicity^43^,^44^. The 250 kDa lower molecular weight hCa_V_3.3 signal decayed relatively rapidly after protein translation was inhibited and was not detectable after 6 hours of puromycin exposure (Fig. 3a). By contrast, the glycosylation-associated >250 kDa hCa_V_3.3 signal decayed more slowly and incompletely (Fig. 3a,b). After 48 hrs of exposure to puromycin, the >250 kDa hCa_V_3.3 signal was reduced ~50% of control levels similarly among three genotypes (Fig. 3b, 48 hr data point, F_(2,20)_ = 2.675, *p* = 0.0935, one-way ANOVA with Dunnett’s post test, *p*_WT, R1346H_ = 0.259, *p*_WT, T797M_ = 0.540). In the first 2 hrs of puromycin treatment, the >250 kDa hCa_V_3.3 signal increased ~40% and ~30% in cells expressing WT and T797M respectively and the levels were not different between the two conditions (Fig. 3b, 2 hr data point, F_(2,20)_ = 17.6, *p* < 0.001, one-way ANOVA with Dunnett’s post test, *p*_WT, T797M_ = 0.859). We observed ~15% reduction in the >250 kDa band in cells expressing R1346H following exposure to puromycin, and the overall protein levels were lower compared to WT (Fig. 3b, 2 hr data point, F_(2,20)_ = 17.6, *p* < 0.001, one-way ANOVA with Dunnett’s post test, *p*_WT, R1346H_ = 0.0154). We also showed that GAPDH levels did not decrease following puromycin treatment in cells expressing different Ca_V_3.3 clones over the time course of the experiment (Fig. 3c). Our data suggest that R1346H reduces the net accumulation of glycosylated hCa_V_3.3 compared to wild-type and does not impact the rates of channel protein decay.

Collectively, our data are consistent with the hypothesis that R1346H interferes with surface trafficking of hCa_V_3.3 by a mechanism that may involve glycosylation. Two putative N-linked glycosylation sites are within 1 and 4 amino acids of R1346 (N1345 and N1342). Mutations of putative glycosylation sites, N192Q and N271Q in S3-S4 and S5-S6 linkers of domain I of Ca_V_3.2 protein are associated with reduced levels of biotinylated, functional channels on the cell membrane surface^45^. The glycosylation states of auxiliary subunits of Ca_V_ channels have also been reported to influence protein function^46^,^47^,^48^.

### hCa_V_3.3 channel current densities are lower in cells expressing R1346H

**Figure 4:**
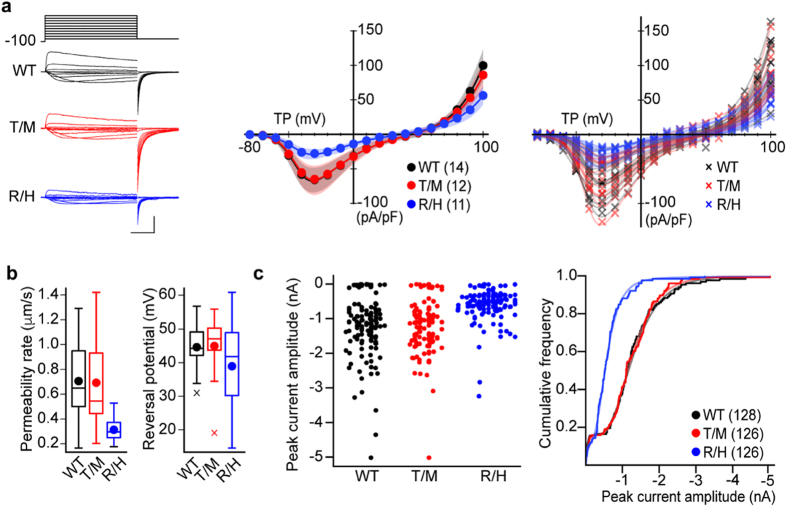
Calcium currents in Flp-In T-Rex HEK293 cells expressing R1346H (R/H) variant are smaller compared to those in cells expressing WT and T797M (T/M). (**a**) *Left*: Calcium currents recorded by whole-cell patch method from three cells expressing WT (black), T/M (red) or R/H (blue) hCa_V_3.3. Currents were evoked by a series of 50 ms long test potentials from a holding potential of −100 mV. Scale bars correspond to 50 pA/pF and 10 ms. *Middle*: Average current-voltage plots. Plots for peak current densities for a range of test potentials (TP) with 99% confidence intervals generated by bootstrap resampling with replacement for cells expressing WT, T/M or R/H hCa_V_3.3. *Right*: Individual current-voltage plots for average data shown in *middle*. (**b**) Average permeability rates (*left*) and reversal potentials (*right*) were estimated from fitting the Goldman-Hodgkin-Katz function to individual current voltage relationships shown in (**a**). Mean (circle), median (horizontal bar), interquartile range (25^th^–75^th^ percentile, box), whiskers (range), and outliers (cross) are shown for each condition. Mean ± SE permeability rates, WT: 0.71 ± 0.08 μm/s (n = 14); T/M: 0.69 ± 0.11 μm/s (n = 12); and R/H: 0.31 ± 0.03 μm/s (n = 11). Mean ± SE reversal potentials, WT: 44.63 ± 2.02 mV (n = 14); T/M: 44.98 ± 2.86 mV (n = 12); and R/H: 38.93 ± 4.27 mV (n = 11). (**c**) Calcium currents recorded by high throughput patch method from Flp-In T-Rex HEK293 cells expressing WT, T/M or R/H hCa_V_3.3. *Left*: Beeswarm plot of peak calcium current amplitudes for each cell line expressing hCa_V_3.3 WT, T/M, and R/H. *Right*: cumulative frequency plot of data shown in left together with fits of each distribution. Median values calculated from parametrization of each distribution, WT: 1.30 nA; T/M: 1.30 nA; and R/H: 0.56 nA. Mean values ± SE, WT: 1.13 ± 0.08 nA (n = 128); T/M: 1.10 ± 0.06 nA (n = 126); and R/H: 0.52 ± 0.04 nA (n = 126).

We next tested if T797M and R1346H affect the functional properties of hCa_V_3.3 channels using whole-cell recording from the same Flp-In T-REx HEK293 cells expressing FLAG-tagged hCa_V_3.3 as described above. We used 2 mM Ca^2+^ as the charge carrier for conventional whole-cell recording and resolved large Ca_V_3.3 currents that peaked at ~60 pA/pF in cells expressing WT channels. Consistent with our biochemical analyses, hCa_V_3.3 channel current densities in doxycycline-induced HEK293 cells expressing R1346H, but not T797M, were smaller relative to those in cells expressing WT hCa_V_3.3, independent of membrane voltage (Fig. 4a). To compare hCa_V_3.3 currents across a range of voltages, we estimated permeability rates from Boltzmann-GHK fits of individual current voltage data sets from cells expressing WT, R1346H and T797M hCa_V_3.3 (Fig. 4b; *left*). Permeability rate was used as a measure of the overall current flow in a cell for a range of voltages (see methods). Permeability rates were ~43.6% lower for hCa_V_3.3 currents in cells expressing R1346H compared to WT (Fig. 4b; *left*, p_WT,R1346H_ = 0.00054, Kolmogorov-Smirnov test followed by Bonferroni correction), whereas permeability rates calculated from cells expressing T797M were similar to WT (Fig. 4b; *left*, *p*_WT,T797M_ = 0.923, Kolmogorov-Smirnov test followed by Bonferroni correction). Estimates of the membrane potential at which Ca_V_3.3 currents reverse direction were not different in cells expressing WT, R1346H and T797M (Fig. 4b; *right*, *p*_WT,R1346H_ = 0.36, *p*_WT,T797M_ = 0.74, Kolmogorov-Smirnov test followed by Bonferroni correction) suggesting that ion selectivity in hCa_V_3.3 channels is unchanged by R1346H and T797M.

We next contracted ChanTest to perform unbiased, high throughput electrophysiology using the Flp-In T-REx HEK293 cells expressing hCa_V_3.3 channels, and data are shown in Fig. 4c (IonWorks Barracuda; Molecular Devices). The large sample sizes, possible from high throughput automated whole-cell analyses, allowed for population analyses of hCav3.3 current densities in cells expressing WT, R1346H or T797M (Fig. 4a,b). Population data, displayed in bee swarm and cumulative frequency plots, illustrate that hCa_V_3.3 currents in cells expressing R1346H hCa_V_3.3 are on average 2-fold smaller compared to WT (Fig. 4c). The cumulative frequency relationships for each condition were bimodal, consistent with two populations of Flp-In T-REx HEK293 cells: one expressing and a smaller fraction not expressing hCa_V_3.3 currents (Fig. 4c). Parameterization of each distribution, allowed by the larger sample size, showed that the percentage of cells not expressing hCa_V_3.3 current was similar among all variants (WT: 16%, T797M: 16%, R1346H: 20%) and that the median hCa_V_3.3 current of cells expressing R1346H was ~2-fold relative to WT (Fig. 4c, *p*_WT,R1346H_ < 0.0001, Kolmogorov-Smirnov test followed by Bonferroni correction). These data are consistent with our findings from conventional whole-cell recording.

**Figure 5:**
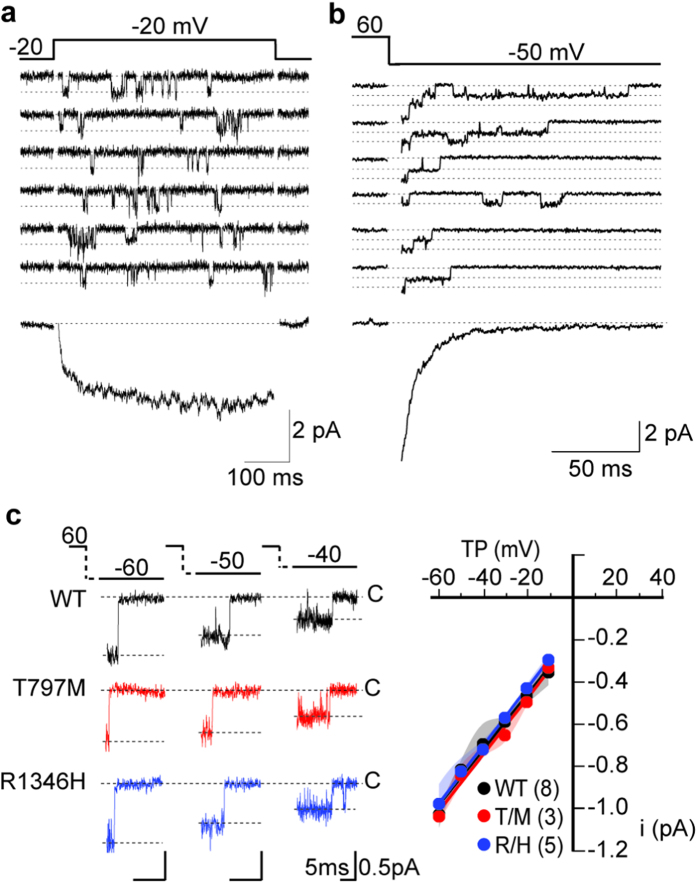
Single hCa_V_3.3 channel currents are unaffected by T797M (T/M) and R1346H (R/H). Recordings are from cell-attached patches from tsA201 cells transiently expressing WT, T/M and R/H hCa_V_3.3. (**a**) Single WT hCa_V_3.3 channel currents evoked by step depolarizations from −80 mV to −20 mV, *upper panel*. Ensemble current trace generated by adding multiple single channel traces recorded at −20 mV, *lower panel*; (**b**) Single hCa_V_3.3 channel tail currents resolved immediately on membrane hyperpolarization to −50 mV from a depolarizing step to +60 mV (used to open the channels), *upper panel*. Tail currents are relatively large because of the large driving force at negative membrane potentials—although they close rapidly. Lower panel shows an ensemble tail current reconstructed from adding multiple single channel tail currents. Closed state is labeled (dotted line). (**c**) Average single Ca_V_3.3 channel current amplitudes at different test potentials (TP, *left panel*) for each clone. Single channel conductances were estimated from slopes of single channel current (i)-voltage relationships (*right panel*). Mean ± SE, WT: 14.0 ± 0.8 pS (n = 8); T/M: 13.3 ± 0.27 pS (n = 3); and R/H: 13.7 ± 0.6 pS (n = 5). In each case, N corresponds to the number of individual patch recordings but each dataset represents measurements of >100 individual channel openings. Averages are shown with 99% confidence intervals calculated using bootstrap with resampling.

Lower hCa_V_3.3 current densities in cells expressing R1346H could originate from fewer hCa_V_3.3 channels on the cell surface, from reduced current flow through individual hCa_V_3.3 channels, when open, or a combination of both. We used high-resolution, low-noise cell-attached patches to measure the rate of ion flow through single hCa_V_3.3 channels directly (Fig. 5a–c). The amplitude of single hCa_V_3.3 channel currents was consistent with that reported previously for single Ca_V_3.3 currents^2^ and indistinguishable among WT, R1346H, and T797M hCa_V_3.3 recordings over a range of test potentials (~13 pS, 110 mM barium as charge carrier; Fig. 5c, *p*_WT,R1346H_ = 0.85, and *p*_WT,T797M_ = 0.53, Kolmogorov-Smirnov test followed by Bonferroni correction). We measured single channel current amplitudes from tail currents to generate the single channel I-V relationship because of the larger current amplitudes (greater driving force) at negative voltages. The slow gating kinetics typical of Ca_V_3.3 currents is illustrated in Fig. 5 from individual traces as well as captured in the ensemble averages. We conclude that the smaller Ca_V_3.3 current densities in cells expressing R1346H reflect reduced numbers of Ca_V_3.3 channels on the cell surface relative to WT, but the amount of current that flows through individual hCa_V_3.3 channels is unaffected by R1346H.

**Figure 6:**
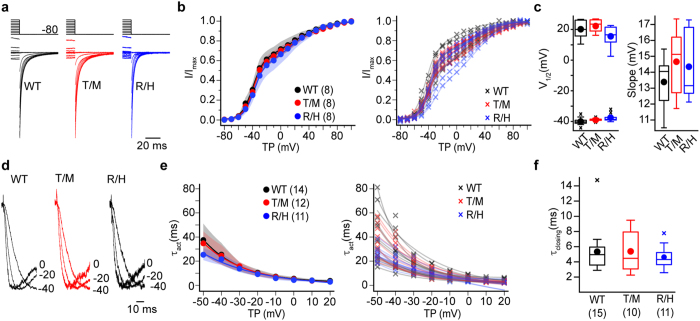
Activation and deactivation properties of Ca_V_3.3 currents in cells expressing WT, T797M (T/M), and R1346H (R/H) hCa_V_3.3 are similar. (**a**) *Left*: hCa_V_3.3 tail currents from cells expressing WT, T/M, and R/H clones. Currents were recorded after membrane potential is hyperpolarized to −80 mV from a series of steps (−80 mV to +60 mV). Current amplitudes were normalized. (**b**) *Left*: I/I_max_ activation at −80 mV from different test potentials. Averages are shown with 99% confidence intervals generated by bootstrap analysis for the three cell lines and; *right*: individual activation curves for each recording. The activation curve is distorted at stronger depolarizations that induce inactivation during the test pulse, but there is no difference among the three clones. (**c**) *Left*: Average V_1/2_ values estimated from fitting two Boltzmann functions to individual activation curves in ***b***. Average (circle), median (horizontal bar), interquartile range (25^th^–75^th^ percentile, box), whiskers (range), and outliers (cross) for each condition. Mean values ± SE for V_1/2-negative_, WT: −40.0 ± 0.98 mV; T/M: −38.8 ± 0.35 mV; and R/H: −37.6 ± 0.99 mV; for V_1/2-positive_, WT: 19.9 ± 2.2 mV; T/M: 22.6 ± 1.46 mV; and R/H: 15.4 ± 2.54 mV; for *k*, WT: 13.4 ± 0.6 mV; T/M: 14.7 ± 0.8 mV; and R/H: 14.3 ± 0.8 mV. (**d**) Calcium currents from cells expressing different hCa_V_3.3 as described above for panels ***a***and ***b***. Left: Currents activated by depolarizations to 0 mV, −20 mV, and −40 mV. Current amplitudes were normalized for visual comparison. (**e**) Time constants estimated from fitting the rising phase of calcium currents evoked by different test potentials (TP) are averaged and plotted as described above for panel***b***. (**f**) Closing rate (τ_closing_) at −60 mV for WT, T/M and R/H hCa_V_3.3 channels. −60 mV was chosen to minimize influence from the differences in current size between R/H and WT. Data are shown similar to panel **b**. Mean values ± SE, τ_closing_ for WT: 5.33 ± 0.75 ms; T/M: 5.38 ± 0.88 ms; and R/H: 4.62 ± 0.46 ms. n values (**b**–**f)**, WT: n = 8; T/M: n = 8; and R/H: n = 8.

**Figure 7:**
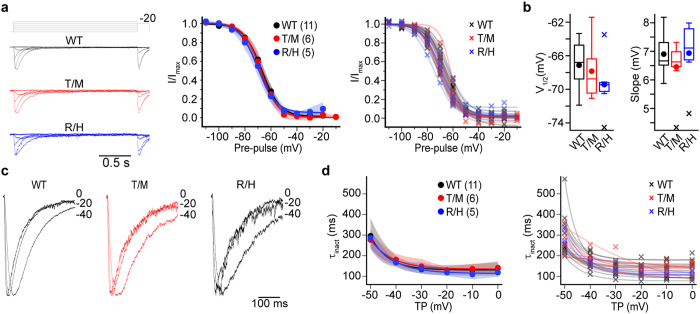
Inactivation properties were similar among WT, T797M (T/M) and R1346H (R/H) hCa_V_3.3 channels. P values calculated using the 2-sample Kolmogorov-Smirnov test (**a**) *Left*: Representative traces to determine the availability of channels to open upon depolarization (voltage-dependence of inactivation). Voltage-dependent inactivation was obtained using a pre-pulse protocol. 2 s inactivating pre-pulses were applied from −110 mV to −10 mV in 10 mV steps; each pre-pulse was followed with a test pulse to −20 mV. *Middle*: voltage dependence of inactivation for WT, T/M and R/H hCav3.3 currents. Symbols represent mean and shaded areas correspond to 95% bootstrapped confidence interval. *Right*: Individual voltage dependent inactivation curves from each genotype are also shown. (**b**) Inactivation curves were fitted to a Boltzmann function. V_1/2_ and slope factor (*k)* were similar among the three genotypes. Average (circle), median (horizontal bar), interquartile range (25^th^–75^th^ percentile, box), whiskers (range), and outliers (cross) are shown for V_1/2_ and *k. Left:* V_1/2_ mean ± SE, WT: −67.1 ± 0.89 mV (n = 11); T/M: −65.3 ± 1.30 mV (n = 6); and R/H: −69.4 ± 1.4 mV (n = 5). *Right*: *k* mean ± SE, WT: 6.90 ± 0.21 mV (n = 11); T/M: 6.45 ± 0.37 mV (n = 6); and R/H: 6.93 ± 0.48 mV (n = 5). (**c**) *Left*: representative traces depicting the rate of decay of the calcium current (open-state inactivation) during the test pulse for WT, T/M, R/H hCa_V_3.3 channels. Traces were normalized to enable comparisons. *Middle*: the decaying phase of calcium currents at several voltages was fitted to a single exponential. The rate of decay increased with test potential depolarization, and the time constants were similar among the three genotypes at all voltages analyzed. *Right*: time constants of inactivation for individual cells.

We completed our assessment of R1346H and T797M hCa_V_3.3 channel properties not captured by analyses of peak current-voltage relationships, using a series of voltage protocols to evaluate whole-cell currents. We assessed: voltage-dependence from tail current analyses (Fig. 6a–c. V_1/2-negative_, *p*_WT,R1346H_ = 0.32, and *p*_WT,T797M_ = 0.91. V_1/2-positive_, *p*_WT,R1346H_ = 0.6, and *p*_WT,T797M_ = 1.00. Slope (*k*), *p*_WT,R1346H_ = 0.52, *p*_WT,T797M_ = 0.19. Kolmogorov-Smirnov test followed by Bonferroni correction), kinetics of channel activation from −50 to 20 mV voltages (Fig. 6d,e), rate of channel closing as derived from the time constant of the tail current decay (Fig. 6f. τ_closing_ at–60 mV, *p*_WT,R1346H_ = 0.43, p_WT,T797M_ = 0.97, Kolmogorov-Smirnov test followed by Bonferroni correction), voltage-dependence of channel inactivation (Fig. 7a,b. V_1/2_, *p*_WT,R1346H_ = 0.12, *p*_WT,T797M_ = 0.76. Slope (*k*), *p*_WT,R1346H_ = 0.52, *p*_WT,T797M_ = 0.19, Kolmogorov-Smirnov test followed by Bonferroni correction), and time course of channel inactivation from −50 to 0 mV (Fig. 7c,d). Tail current kinetics provide a measure of the overall rate of channel closing, because the tail potential is below the threshold for channel opening and the slow closing kinetics is a hallmark feature of Ca_V_3 channels, compared to other Ca_V_ channels. We conclude from these extensive analyses, that R1346H and T797M do not affect the biophysical properties of hCa_V_3.3 channels as assessed in human cell lines, including the time course of recovery from inactivation (T797M; data not shown). It is important to note that our analyses, which find T797M has no measurable effect on the basic features of hCa_V_3.3 channels including expression levels and biophysical properties, do not rule out a potential effect of T797M that depends on the presence of cofactors in the native environment.

### R1346H impacts rebound bursting in a model of TRN neuron

**Figure 8:**
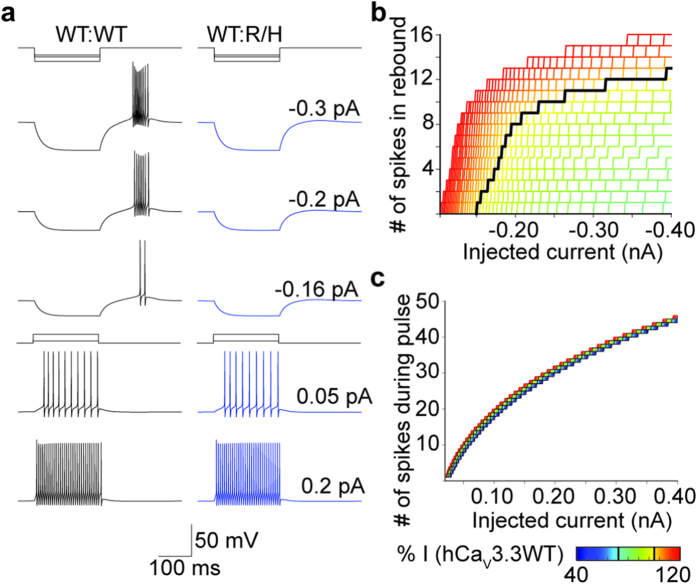
hCa_V_3.3 R1346H (R/H) variant impairs rebound burst firing in TRN neuron simulations. (**a**) Simulations of membrane voltage in model TRN neurons responding to hyperpolarizing or depolarizing current injections. *Left*: WT neuron fires a burst of rebound action potentials following 200 ms negative, hyperpolarizing current injections of different magnitudes (upper) and bursts of action potentials during the depolarizing current injection (lower). *Right*: Neuron with 72.5% of WT Ca_V_3.3 current density to approximate heterozygosity (50% R/H and 50% WT) does not exhibit rebound bursting following hyperpolarization. (**b**) Relationship between number of rebound spikes and size of hyperpolarizing current injection for a range of Ca_V_3.3 current densities. Percent of Ca_V_3.3 current relative to WT (black) color coded from green (78%) to red (120%). (**c**) The number of spikes during positive current injections depends on the magnitude of the current injection (x-axis), but it is only marginally influenced by Ca_V_3.3 density. Black lines represent current density for WT and R/H heterozygous cells, with same color spectrum as in (**b**), extended to 40% of WT (blue).

In TRN neurons, transient membrane hyperpolarizations can trigger bursts of action potentials, called rebound bursting, immediately upon membrane repolarization^7^,^8^ (Fig. 8a). This phenomenon is mediated by activation of dendritic Ca_V_3.3 channels that are recruited from a previously inactivated state when the membrane is hyperpolarized. Ca_V_3.3 underlies 90% of the low threshold, voltage-gated calcium channel expressed in TRN^9^ and rebound bursting is absent in TRN neurons of mice lacking Ca_V_3.3^6^,^9^. By simulating TRN neuron excitability in the NEURON environment^7^ we show that: (i) rebound bursting is highly sensitive to Ca_V_3.3 channel density; (ii) rebound bursting is eliminated when Ca_V_3.3 channel density is reduced to 78% or less of initial WT values (Fig. 8b; black line shows WT relationship); and (iii) firing of TRN neurons evoked by depolarizing current injections is insensitive to changes in dendritic Ca_V_3.3 current densities to 40% of initial WT values (Fig. 8a,c).

To simulate heterozygosity—equal contribution of WT and R1346H alleles—we reduced Ca_V_3.3 current density to 72.5% of WT levels and showed that this reduction in Ca_V_3.3 current density fails to support rebound bursting regardless of hyperpolarization magnitude (Fig. 8a, top three rows). In contrast, firing of TRN neurons, evoked by depolarizing current injections is unaffected by R1346H (Fig. 8a, bottom two rows). The results from NEURON simulation are consistent with the notion that Depolarization-induced firing is primarily mediated by activation of voltage-gated ion channels other than Ca_V_3.3 in TRN^8^,^9^.

## Discussion

*CACNA1I* has been identified as a candidate schizophrenia risk gene based on genome wide association studies, and on the identification of *de novo*, rare missense variations in *CACNA1I* from exome sequencing of schizophrenia proband trios^19^,^31^. Our study is the first to assess the functional impact of two missense *CACNA1I* variants found in schizophrenia patients, but not in unaffected family members^31^. Based on our analyses, we find that the *de novo* coding variant Chr22: 39665939G > A of *CACNA1I*^31^, is sufficiently disruptive to Ca_V_3.3 in the heterozygous condition to impact rebound bursting in a TRN model neuron. Our data lend support to the proposal that Ca_V_3.3 R1346H, which is proposed as one of several *de novo* risk variations that contribute to schizophrenia pathophysiology^31^, is damaging. We did not find any evidence that the *de novo* coding variation Chr22: 39659492C > T of *CACNA1I* (T797M Ca_V_3.3) is disruptive in biochemical and electrophysiological HEK cell assays. However, we cannot rule out the possibility that T797M impacts Ca_V_3.3 channel function by mechanisms that are not reconstituted in HEK cells.

Our findings are interesting in light of documented functional associations between reduced Ca_V_3.3 expression in TRN neurons, rebound bursting of TRN neurons, sleep spindle oscillations, and sleep spindle coherence across cortex in schizophrenia^6^,^10^,^14^,^49^,^50^,^51^. Moreover, reduced spindle activity is a heritable component of the sleep electroencephalogram patterns detected in the 1^st^ degree relatives of people with schizophrenia^52^.

Our data are consistent with a mechanism by which R1346H interferes with Ca_V_3.3 glycosylation and plasma membrane trafficking leading to reduced Ca_V_3.3 current density. An extracellular N-linked glycosylation motif N1345 (N-x-S/T) encompasses R1346 residue and glycosylation of proteins in the endoplasmic reticulum (ER) is known to influence the rate of protein transport from the ER through the Golgi apparatus to the plasma membrane^53^. Our studies add to reports that glycosylation of many ion channel proteins, including Ca_V_3.2, regulates their levels of surface expression^45^,^54^,^55^,^56^,^57^,^58^. Several human diseases are known to arise from defects in glycosylation. For example, in cystic fibrosis causal mutations in human *CFTR* lead to altered glycosylation patterns of CFTR channel protein and channel trafficking defects in lung epithelial cells, and in long QT syndrome, coding mutations in human *KCNE* and *KCNQ* lead to reduced potassium ion channel glycosylation and reduced expression in the heart^59^,^60^,^61^,^62^.

Ca_V_3.3 is essential for rebound bursting in TRN neurons, and we show that reduced Ca_V_3.3 current density in model TRN neurons expressing R1346H is sufficient to disrupt this phenomenon. Ca_V_3.3 is expressed in other brain regions including the cortex and in mitral cell dendrites of the olfactory bulb^63^. In mitral cells, Ca_V_3.3 contributes to modulation of evoked and asynchronous release, and it mediates rebound bursting^63^. Deficits in olfaction have, for several years, been described in people with schizophrenia^64^.

It is widely accepted that schizophrenia disease risk depends on the accumulated effect of multiple or many common risk loci^19^, but the relative contribution of each individual rare variant to the disease risk is not known^17^. Rare, coding variations have the greatest potential to disrupt protein function and are likely to make greater contribution to complex common disease risk including schizophrenia^17^,^19^,^25^,^30^,^65^. Our experiments were designed to assess the functional consequences of T797M and R1346H on Ca_V_3.3 channel activity in a robust expression system, and they demonstrate that R1346H disrupts Ca_V_3.3 channel trafficking to the plasma membrane. We did not identify a phenotype associated with T797M in our assays of Ca_V_3.3 function and, as discussed above, we cannot rule out the possibility that T797M will be disruptive to Ca_V_3.3 signaling in the native environment, but our analyses should help guide future studies designed to assess the potential contribution of R1346H-Ca_V_3.3 to schizophrenia risk.

## Additional Information

**How to cite this article**: Andrade, A. *et al.* A rare schizophrenia risk variant of *CACNA1I* disrupts Ca_V_3.3 channel activity. *Sci. Rep.* **6**, 34233; doi: 10.1038/srep34233 (2016).

## References

[b1] Perez-Reyes E. Molecular physiology of low-voltage-activated t-type calcium channels. Physiol Rev 83, 117–161 (2003). 12506128 10.1152/physrev.00018.2002

[b2] Lee J. H. *et al.* Cloning and expression of a novel member of the low voltage-activated T-type calcium channel family. J Neurosci 19, 1912–1921 (1999). 10066244 10.1523/JNEUROSCI.19-06-01912.1999 PMC6782566

[b3] Houser C. R., Vaughn J. E., Barber R. P. & Roberts E. GABA neurons are the major cell type of the nucleus reticularis thalami. Brain Res 200, 341–354 (1980). 7417821 10.1016/0006-8993(80)90925-7

[b4] Liu X. B., Murray K. D. & Jones E. G. Low-threshold calcium channel subunit Ca(v) 3.3 is specifically localized in GABAergic neurons of rodent thalamus and cerebral cortex. J Comp Neurol 519, 1181–1195 (2011). 21344408 10.1002/cne.22567

[b5] Steriade M. Grouping of brain rhythms in corticothalamic systems. Neuroscience 137, 1087–1106 (2006). 16343791 10.1016/j.neuroscience.2005.10.029

[b6] Astori S. *et al.* The Ca(V)3.3 calcium channel is the major sleep spindle pacemaker in thalamus. Proc Natl Acad Sci USA 108, 13823–13828 (2011). 21808016 10.1073/pnas.1105115108 PMC3158184

[b7] Destexhe A., Contreras D., Steriade M., Sejnowski T. J. & Huguenard J. R. *In vivo*, *in vitro*, and computational analysis of dendritic calcium currents in thalamic reticular neurons. J Neurosci 16, 169–185 (1996). 8613783 10.1523/JNEUROSCI.16-01-00169.1996 PMC6578708

[b8] Huguenard J. R. & Prince D. A. Intrathalamic rhythmicity studied *in vitro*: nominal T-current modulation causes robust antioscillatory effects. J Neurosci 14, 5485–5502 (1994). 8083749 10.1523/JNEUROSCI.14-09-05485.1994 PMC6577071

[b9] Lee S. E. *et al.* Rebound burst firing in the reticular thalamus is not essential for pharmacological absence seizures in mice. Proc Natl Acad Sci USA 111, 11828–11833 (2014). 25071191 10.1073/pnas.1408609111 PMC4136605

[b10] Ferrarelli F. & Tononi G. The thalamic reticular nucleus and schizophrenia. Schizophr Bull 37, 306–315 (2011). 21131368 10.1093/schbul/sbq142 PMC3044616

[b11] Pinault D. Dysfunctional thalamus-related networks in schizophrenia. Schizophr Bull 37, 238–243 (2011). 21307040 10.1093/schbul/sbq165 PMC3044615

[b12] Pratt J. A. & Morris B. J. The thalamic reticular nucleus: a functional hub for thalamocortical network dysfunction in schizophrenia and a target for drug discovery. J Psychopharmacol 29, 127–137 (2015). 25586397 10.1177/0269881114565805

[b13] Vukadinovic Z. Sleep abnormalities in schizophrenia may suggest impaired trans-thalamic cortico-cortical communication: towards a dynamic model of the illness. Eur J Neurosci 34, 1031–1039 (2011). 21895800 10.1111/j.1460-9568.2011.07822.x

[b14] Wamsley E. J. *et al.* Reduced sleep spindles and spindle coherence in schizophrenia: mechanisms of impaired memory consolidation. Biol Psychiatry 71, 154–161 (2012). 21967958 10.1016/j.biopsych.2011.08.008 PMC3561714

[b15] Cross-Disorder G. O. T. P. G. C. Identification of risk loci with shared effects on five major psychiatric disorders: a genome-wide analysis. Lancet 381, 1371–1379 (2013). 23453885 10.1016/S0140-6736(12)62129-1 PMC3714010

[b16] Fromer M. *et al.* *De novo* mutations in schizophrenia implicate synaptic networks. Nature 506, 179–184 (2014). 24463507 10.1038/nature12929 PMC4237002

[b17] Purcell S. M. *et al.* A polygenic burden of rare disruptive mutations in schizophrenia. Nature 506, 185–190 (2014). 24463508 10.1038/nature12975 PMC4136494

[b18] Samocha K. E. *et al.* A framework for the interpretation of de novo mutation in human disease. Nat Genet 46, 944–950 (2014). 25086666 10.1038/ng.3050 PMC4222185

[b19] Schizophrenia W. G. O. T. P. G. C. Biological insights from 108 schizophrenia-associated genetic loci. Nature 511, 421–427 (2014). 25056061 10.1038/nature13595 PMC4112379

[b20] Curtis D. *et al.* Case-case genome-wide association analysis shows markers differentially associated with schizophrenia and bipolar disorder and implicates calcium channel genes. Psychiatr Genet 21, 1–4 (2011). 21057379 10.1097/YPG.0b013e3283413382 PMC3024533

[b21] Heyes S. *et al.* Genetic disruption of voltage-gated calcium channels in psychiatric and neurological disorders. Prog Neurobiol 134, 36–54 (2015). 26386135 10.1016/j.pneurobio.2015.09.002 PMC4658333

[b22] Moskvina V. *et al.* Gene-wide analyses of genome-wide association data sets: evidence for multiple common risk alleles for schizophrenia and bipolar disorder and for overlap in genetic risk. Mol Psychiatry 14, 252–260 (2009). 19065143 10.1038/mp.2008.133 PMC3970088

[b23] Zamponi G. W. Targeting voltage-gated calcium channels in neurological and psychiatric diseases. Nat Rev Drug Discov 15, 19–34 (2016). 26542451 10.1038/nrd.2015.5

[b24] International S. C. *et al.* Common polygenic variation contributes to risk of schizophrenia and bipolar disorder. Nature 460, 748–752 (2009). 19571811 10.1038/nature08185 PMC3912837

[b25] Heinzen E. L., Neale B. M., Traynelis S. F., Allen A. S. & Goldstein D. B. The genetics of neuropsychiatric diseases: looking in and beyond the exome. Annu Rev Neurosci 38, 47–68 (2015). 25840007 10.1146/annurev-neuro-071714-034136

[b26] Limpitikul W. B., Dick I. E., Ben-Johny M. & Yue D. T. An autism-associated mutation in CaV1.3 channels has opposing effects on voltage- and Ca(2+)-dependent regulation. Sci Rep 6, 27235 (2016). 27255217 10.1038/srep27235 PMC4891671

[b27] Pinggera A. *et al.* CACNA1D *de novo* mutations in autism spectrum disorders activate Cav1.3 L-type calcium channels. Biol Psychiatry 77, 816–822 (2015). 25620733 10.1016/j.biopsych.2014.11.020 PMC4401440

[b28] Roussos P. *et al.* A role for noncoding variation in schizophrenia. Cell Rep 9, 1417–1429 (2014). 25453756 10.1016/j.celrep.2014.10.015 PMC4255904

[b29] Yoshimizu T. *et al.* Functional implications of a psychiatric risk variant within CACNA1C in induced human neurons. Mol Psychiatry 20, 162–169 (2015). 25403839 10.1038/mp.2014.143 PMC4394050

[b30] Zhang X., Bailey S. D. & Lupien M. Laying a solid foundation for Manhattan–’setting the functional basis for the post-GWAS era’. Trends Genet 30, 140–149 (2014). 24661571 10.1016/j.tig.2014.02.006 PMC4026049

[b31] Gulsuner S. *et al.* Spatial and temporal mapping of *de novo* mutations in schizophrenia to a fetal prefrontal cortical network. Cell 154, 518–529 (2013). 23911319 10.1016/j.cell.2013.06.049 PMC3894107

[b32] Schneider-Poetsch T. *et al.* Inhibition of eukaryotic translation elongation by cycloheximide and lactimidomycin. Nat Chem Biol 6, 209–217 (2010). 20118940 10.1038/nchembio.304 PMC2831214

[b33] Livak K. J. & Schmittgen T. D. Analysis of relative gene expression data using real-time quantitative PCR and the 2(-Delta Delta C(T)) Method. Methods 25, 402–408 (2001). 11846609 10.1006/meth.2001.1262

[b34] Pan J. Q. *et al.* AKT kinase activity is required for lithium to modulate mood-related behaviors in mice. Neuropsychopharmacology 36, 1397–1411 (2011). 21389981 10.1038/npp.2011.24 PMC3096809

[b35] Groen J. L. *et al.* CACNA1B mutation is linked to unique myoclonus-dystonia syndrome. Hum Mol Genet 24, 987–993 (2015). 25296916 10.1093/hmg/ddu513 PMC4817404

[b36] Raingo J., Castiglioni A. J. & Lipscombe D. Alternative splicing controls G protein-dependent inhibition of N-type calcium channels in nociceptors. Nat Neurosci 10, 285–292 (2007). 17293861 10.1038/nn1848 PMC3027493

[b37] Castiglioni A. J., Raingo J. & Lipscombe D. Alternative splicing in the C-terminus of CaV2.2 controls expression and gating of N-type calcium channels. J Physiol 576, 119–134 (2006). 16857708 10.1113/jphysiol.2006.115030 PMC1995641

[b38] Lipscombe D. *et al.* Spatial distribution of calcium channels and cytosolic calcium transients in growth cones and cell bodies of sympathetic neurons. Proc Natl Acad Sci USA 85, 2398–2402 (1988). 2451249 10.1073/pnas.85.7.2398 PMC280000

[b39] Lipscombe D., Kongsamut S. & Tsien R. W. Alpha-adrenergic inhibition of sympathetic neurotransmitter release mediated by modulation of N-type calcium-channel gating. Nature 340, 639–642 (1989). 2570354 10.1038/340639a0

[b40] Gomora J. C., Murbartián J., Arias J. M., Lee J. H. & Perez-Reyes E. Cloning and expression of the human T-type channel Ca(v)3.3: insights into prepulse facilitation. Biophys J 83, 229–241 (2002). 12080115 10.1016/s0006-3495(02)75164-3 PMC1302142

[b41] Kuryshev Y. A., Brown A. M., Duzic E. & Kirsch G. E. Evaluating state dependence and subtype selectivity of calcium channel modulators in automated electrophysiology assays. Assay Drug Dev Technol 12, 110–119 (2014). 24579774 10.1089/adt.2013.552 PMC4657039

[b42] Migliore M. *et al.* ModelDB: making models publicly accessible to support computational neuroscience. Neuroinformatics 1, 135–139 (2003). 15055399 10.1385/NI:1:1:135 PMC3728921

[b43] Mbonye U. R. *et al.* Two distinct pathways for cyclooxygenase-2 protein degradation. J Biol Chem 283, 8611–8623 (2008). 18203712 10.1074/jbc.M710137200

[b44] Tcherkezian J. *et al.* Proteomic analysis of cap-dependent translation identifies LARP1 as a key regulator of 5′TOP mRNA translation. Genes Dev 28, 357–371 (2014). 24532714 10.1101/gad.231407.113 PMC3937514

[b45] Weiss N., Black S. A., Bladen C., Chen L. & Zamponi G. W. Surface expression and function of Cav3.2 T-type calcium channels are controlled by asparagine-linked glycosylation. Pflugers Arch 465, 1159–1170 (2013). 23503728 10.1007/s00424-013-1259-3

[b46] Gurnett C. A., De Waard M. & Campbell K. P. Dual function of the voltage-dependent Ca^2+^ channel alpha 2 delta subunit in current stimulation and subunit interaction. Neuron 16, 431–440 (1996). 8789958 10.1016/s0896-6273(00)80061-6

[b47] Price M. G., Davis C. F., Deng F. & Burgess D. L. The alpha-amino-3-hydroxyl-5-methyl-4-isoxazolepropionate receptor trafficking regulator “stargazin” is related to the claudin family of proteins by Its ability to mediate cell-cell adhesion. J Biol Chem 280, 19711–19720 (2005). 15760900 10.1074/jbc.M500623200 PMC1255971

[b48] Sandoval A., Oviedo N., Andrade A. & Felix R. Glycosylation of asparagines 136 and 184 is necessary for the alpha2delta subunit-mediated regulation of voltage-gated Ca^2+^ channels. FEBS Lett 576, 21–26 (2004). 15474003 10.1016/j.febslet.2004.08.054

[b49] Ferrarelli F. *et al.* Thalamic dysfunction in schizophrenia suggested by whole-night deficits in slow and fast spindles. Am J Psychiatry 167, 1339–1348 (2010). 20843876 10.1176/appi.ajp.2010.09121731 PMC2970761

[b50] Göder R. *et al.* Impairment of sleep-related memory consolidation in schizophrenia: relevance of sleep spindles. Sleep Med 16, 564–569 (2015). 25912599 10.1016/j.sleep.2014.12.022

[b51] Manoach D. S. *et al.* Reduced overnight consolidation of procedural learning in chronic medicated schizophrenia is related to specific sleep stages. J Psychiatr Res 44, 112–120 (2010). 19665729 10.1016/j.jpsychires.2009.06.011 PMC2813320

[b52] Manoach D. S. *et al.* Sleep spindle deficits in antipsychotic-naïve early course schizophrenia and in non-psychotic first-degree relatives. Front Hum Neurosci 8, 762 (2014). 25339881 10.3389/fnhum.2014.00762 PMC4188028

[b53] Lodish H. *et al.* Molecular Cell Biology. 4th Edition. (W.H. Freeman, New York, 2000).

[b54] Estévez R. & Jentsch T. J. CLC chloride channels: correlating structure with function. Curr Opin Struct Biol 12, 531–539 (2002). 12163078 10.1016/s0959-440x(02)00358-5

[b55] Gong Q., Anderson C. L., January C. T. & Zhou Z. Role of glycosylation in cell surface expression and stability of HERG potassium channels. Am J Physiol Heart Circ Physiol 283, H77–H84 (2002). 12063277 10.1152/ajpheart.00008.2002

[b56] Khanna R., Myers M. P., Lainé M. & Papazian D. M. Glycosylation increases potassium channel stability and surface expression in mammalian cells. J Biol Chem 276, 34028–34034 (2001). 11427541 10.1074/jbc.M105248200

[b57] Riordan J. R. Assembly of functional CFTR chloride channels. Annu Rev Physiol 67, 701–718 (2005). 15709975 10.1146/annurev.physiol.67.032003.154107

[b58] Shi G. *et al.* Beta subunits promote K^+^ channel surface expression through effects early in biosynthesis. Neuron 16, 843–852 (1996). 8608002 10.1016/s0896-6273(00)80104-x

[b59] Akhavan A. N- and O-linked glycosylation coordinate cell-surface localization of a cardiac potassium channel. J Physiol 589, 4647–4648 (2011). 21965632 10.1113/jphysiol.2011.218263 PMC3213413

[b60] Ntimbane T. *et al.* Cystic fibrosis-related diabetes: from CFTR dysfunction to oxidative stress. Clin Biochem Rev 30, 153–177 (2009). 20011209 PMC2791770

[b61] O’Riordan C. R., Lachapelle A. L., Marshall J., Higgins E. A. & Cheng S. H. Characterization of the oligosaccharide structures associated with the cystic fibrosis transmembrane conductance regulator. Glycobiology 10, 1225–1233 (2000). 11087715 10.1093/glycob/10.11.1225

[b62] Smith J. L. *et al.* Trafficking-deficient hERG K^+^channels linked to long QT syndrome are regulated by a microtubule-dependent quality control compartment in the ER. Am J Physiol Cell Physiol 301, C75–C85 (2011). 21490315 10.1152/ajpcell.00494.2010 PMC3129823

[b63] Fekete A., Johnston J. & Delaney K. R. Presynaptic T-type Ca^2+^ channels modulate dendrodendritic mitral-mitral and mitral-periglomerular connections in mouse olfactory bulb. J Neurosci 34, 14032–14045 (2014). 25319700 10.1523/JNEUROSCI.0905-14.2014 PMC6705286

[b64] Kopala L. C., Good K. P., Torrey E. F. & Honer W. G. Olfactory function in monozygotic twins discordant for schizophrenia. Am J Psychiatry 155, 134–136 (1998). 9433354 10.1176/ajp.155.1.134

[b65] Zuk O. *et al.* Searching for missing heritability: designing rare variant association studies. Proc Natl Acad Sci USA 111, E455–E464 (2014). 24443550 10.1073/pnas.1322563111 PMC3910587

